# What Does N2 Lymph Node Involvement Mean for Patients with Non-Small Cell Lung Cancer (NSCLC)?—A Review of Implications for Diagnosis and Treatment

**DOI:** 10.3390/cancers16152673

**Published:** 2024-07-26

**Authors:** Julio Linares Díaz, John Edwards, Anne-Leen Deleu, Niccolo Giaj-Levra, Elena Prisciandaro, Benoit Roch, Marianne Paesmans, Thierry Berghmans, Mariana Brandão

**Affiliations:** 1 Medical Oncology Department, Hospital La Fe, Avinguda de Fernando Abril Martorell 106, 46006 Valencia, Spain; julio_mdps@hotmail.com; 2 Thoracic Oncology Functional Unit, Institut Jules Bordet, Hôpital Universitaire de Bruxelles, Rue Meylemeersch 90, 1070 Brussels, Belgium; thierry.berghmans@hubruxelles.be; 3 Department of Cardiothoracic Surgery, Sheffield Teaching Hospitals NHS Foundation Trust, Herries Road, Sheffield S5 5AU, UK; john.edwards3@nhs.net; 4 Nuclear Medicine Department, Institut Jules Bordet, Hôpital Universitaire de Bruxelles, Rue Meylemeersch 90, 1070 Brussels, Belgium; anne-leen.deleu@hubruxelles.be; 5 Advanced Radiation Oncology Department, IRCCS Sacro Cuore Don Calabria Hospital, 37024 Verona, Italy; niccolo.giajlevra@sacrocuore.it; 6 Department of Thoracic Surgery, Hôpital Erasme, Hôpital Universitaire de Bruxelles, Route de Lennik 808, 1070 Brussels, Belgium; elena.prisciandaro@hubruxelles.be; 7 Thoracic Oncology Unit, Department of Respiratory Diseases, University of Montpellier, CHU Montpellier, F-34295 Montpellier, France; benoit2.roch@inserm.fr; 8 Montpellier Cancer Research Institute, University of Montpellier, ICM, INSERM, F-34298 Montpellier, France; 9 Statistics Department, Institut Jules Bordet, Hôpital Universitaire de Bruxelles, Rue Meylemeersch 90, 1070 Brussels, Belgium; marianne.paesmans@hubruxelles.be

**Keywords:** lung cancer, stage III, N2, treatment, staging

## Abstract

**Simple Summary:**

Approximately one third of patients with non-small cell lung cancer are diagnosed with N2 disease corresponding to stages IIIA–IIIB, according to the current 8th TNM edition. The management of these tumors typically involves a combination of treatment modalities including surgery, radiotherapy, chemotherapy, immunotherapy, and targeted therapy. However, N2 disease is highly heterogeneous and may be subcategorized into “single-station”, “multi-station”, “bulky”, and “invasive”, leading to different diagnostic, staging, and treatment approaches. Staging definitions are currently being reevaluated due to the complex nature of N2 disease and its prognostic implications. Diagnostic methods range from imaging studies to minimally invasive approaches (i.e., endoscopy) to more invasive surgical procedures (i.e., mediastinoscopy). Treatment options include surgery with or without additional perioperative treatments as well as definitive chemoradiotherapy for patients with unresectable tumors. Therefore, given this diversity, it is crucial for healthcare professionals to develop management strategies tailored on individual N2 patient characteristics.

**Abstract:**

Patients with stage III NSCLC with N2 lymph node involvement carry a complex and diverse disease entity. Challenges persist in the areas of diagnosis, staging, multimodal management, and the determination of surgical indications and resectability criteria. Therefore, this review focuses on the latest updates in N2 disease staging and its prognostic and treatment implications. Emphasis is placed on the importance of accurate staging using imaging modalities such as [18F]FDG-PET/CT as well as minimally invasive mediastinal staging endoscopic techniques. The evolving role of surgery in the management of N2 disease is also explored. The benefits of neoadjuvant and adjuvant treatments have been demonstrated, along with the efficacy of a combined multimodal approach with chemo-immunotherapy in the perioperative setting, reigniting the debate of N2 disease subsets and optimal treatment options. Furthermore, this review addresses the controversies surrounding surgical approaches in upfront “borderline” resectable stage III NSCLC as well as the benefits of combined chemoradiotherapy with consolidation immunotherapy for patients with unresectable tumors. In conclusion, personalized diagnostic and treatment approaches tailored to individual patient characteristics, resource availability, and institutional expertise are essential for optimizing outcomes in patients with stage III-N2 NSCLC.

## 1. Introduction

Around 20–35% of patients with non-small cell lung cancer (NSCLC) are diagnosed with stage III disease [1]. This stage comprises tumors with a wide range of sizes and locations (T1–T4) as well as lymph node involvement (N0–N3). Specifically, patients with stage III-N2 tumors (corresponding to stages IIIA–IIIB) represent 15–20% of all NSCLC patients [2,3]. The management of these tumors usually combines multiple treatment modalities such as surgery, radiotherapy, chemotherapy, and more recently, immunotherapy and targeted therapy [4].

The recent trials showing the remarkable survival benefit of using immunotherapy in the peri-operative setting for resectable tumors have reignited the debate about the definition of the different N2 disease categories, the diagnostic modalities that should be used to prove it, and the best treatment options for each subset of patients [5,6,7,8,9]. Therefore, with this narrative review, we aim to shed light on these many open questions.

## 2. Definition of N2 Disease

Cancer staging provides a standardized classification system used to describe the anatomical extent of malignancy and its relationship with prognosis, also serving as a common language for discussing treatment options. Systematic staging is composed of three categories: tumor (T), lymph node involvement (N), and distant metastasis (M). For lung cancer staging, the N factor has historically been graded based on anatomical location, and traditionally does not include the quantification of the involved lymph nodes [10].

According to the current 8th TNM staging edition for NSCLC, N2 involvement is defined as metastasis in ipsilateral mediastinal and/or subcarinal lymph node(s) [11]. This involvement comprises nodes: (i) in the superior mediastinal zone including stations 2R (upper paratracheal right), 2L (upper paratracheal left), 3a (prevascular), 3p (retrotracheal), 4R (lower paratracheal right), and 4L (lower paratracheal left); (ii) in the inferior mediastinal zone, subdivided in the subcarinal zone (including station 7, subcarinal), and the inferior mediastinal lower zone including stations 8 (paraesophageal, below carina) and 9 (pulmonary ligament) (iii) in the aortopulmonary zone including stations 5 (aortopulmonary window) and 6 (para-aortic nodes).

Based on the number of stations infiltrated by tumor metastases, N2 can be subdivided into single-station, multiple-station but limited to one zone, and multiple-station in multiple zones [12]. Furthermore, N2 involvement can be classified according to its size as “bulky” or “non-bulky”, but there is not a single definition of “bulky” in the literature. International guidelines describe “non-bulky” lymph nodes as “easily measurable lymph node, whose short-axis diameter is less than 3 cm, and that are free of major mediastinal structures including the trachea and great vessels” [13]. However, other authors consider bulky N2 disease as lymph nodes larger than 2.5 cm diameter in the short axis [14]. It should be noted that the current 8th TNM staging edition does not describe “bulky” N2 disease as a distinct subgroup nor it is expected to become one separate subgroup in the upcoming 9th edition [10,11]. Another important descriptor of N2 disease is its “invasiveness”: N2 invasive lymph nodes are those considered to invade nearby structures [15].

**Figure 1:**
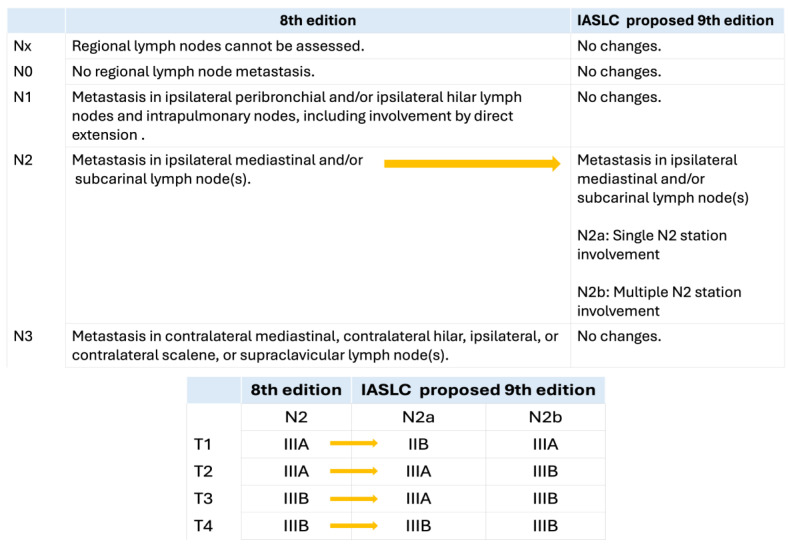
Proposed changes of the N descriptors for the upcoming 9th edition of the TNM classification by the International Association for the Study of Lung Cancer (IASLC) and its implications in tumor stages.

Thus, not surprisingly, given the heterogeneity of N2 involvement, the International Association for the Study of Lung Cancer (IASLC) proposed a revision of the N descriptors in preparation for the upcoming 9th edition of the TNM classification [10]. Exploratory analysis of lymph node subclassification proposed in the 8th TNM edition by Asamura et al. did not provide a clear and consistent separation between subcategories across clinical and pathologic stages [16]. Therefore, a simpler proposal was developed that only divided N2 as single-station (N2a) or multiple-station (N2b) involvement (Figure 1). The distinction in terms of prognosis between single-station N2 and multiple-station N2 remained consistent across various factors such as histologic type, resection status, geographic region, and T category [10]. Thus, the emphasis has moved away from the previously proposed “zones” to lymph node “stations”, which places the onus on the clinical team to recognize the anatomical boundaries of lymph node stations, in order to derive accurate preoperative and intraoperative staging.

This change also means that, while in the 8th TNM edition, all tumors with N2 involvement are classified into stages IIIA and IIIB, in the upcoming 9th edition, the T1N2a tumors will be “downstaged” to stage IIB, while the rest of the N2 tumors will remain in the stage IIIA/B category (Figure 1).

## 3. Implications for Diagnosis

### 3.1. Imaging Diagnosis

International guidelines recommend that the initial staging of NSCLC should include a contrast enhanced computed tomography (CT) scan of the chest and upper abdomen including the liver and the adrenal glands [17]. Moreover, ^18^F-fluorodeoxyglucose (FDG) positron emission tomography/computed tomography (PET/CT), a noninvasive imaging method that integrates the metabolic functional information provided by PET with the anatomic information provided by CT, is being increasingly used for nodal staging. A systematic review and meta-analysis showed high sensitivity and specificity estimates for malignant lymph node detection on FDG PET/CT by using either an absolute SUV threshold (SUVmax 2.5) or a relative one (lymph node versus background uptake) [18]. However, the semi-quantitative analysis of FDG PET/CT should be interpreted with caution given the fact that the patient’s comorbidities (such as history of granulomatous disease) and PET acquisition protocols can largely influence the SUV measurements [19]. Therefore, CT findings on PET/CT images such as benign calcifications or attenuation in terms of Hounsfield units (HU) should be used to differentiate inflammatory from malignant hypermetabolic lymph nodes [18].

In studies in which a node SUVmax ≥ 2.5 on PET/CT was the sole criterion for the detection of metastatic lymph nodes, the sensitivity and specificity estimates for this threshold were 81.3% (95% confidence intervals [CI], 70.2–88.9) and 79.4% (95% CI, 70–86.5), respectively [18]. This means that PET/CT alone is not enough to rule out or confirm a neoplastic mediastinal invasion. Moreover, such a threshold is imperfect as the SUVmax is highly influenced by numerous factors such as the patient’s glycemia and should always be reported to mediastinal background noise.

Thus, if the PET/CT scan shows positive lymph nodes in the mediastinum, this lymph node status should be pathologically confirmed. A phenomenon of bilateral diffuse FDG activity in multiple thoracic lymph nodes is often seen in patients with chronic inflammation from conditions such as pneumonitis or anthracosis, which may be mistakenly interpreted as cancer involvement and significantly impact the treatment approach for these patients [20].

### 3.2. Invasive Diagnosis

The role of invasive mediastinal nodal staging (IMNS) in patients without radiologic evidence of lymph node metastasis remains inconclusive. A retrospective cohort study conducted by Kim et al. examined the impact of IMNS on long-term survival in 4545 patients lacking evidence of lymph node metastasis on imaging studies. Approximately 20% of the patients underwent IMNS. When analyzing the data and adjusting for confounding variables such as sex, performance status, and tumor FDG avidity, the study found no significant difference in overall survival (OS) and disease-free survival (DFS) between patients who underwent IMNS and those who did not. However, this study did not differentiate between mediastinoscopy and EBUS as methods of IMNS, failing to reflect the differences in invasiveness and diagnostic yield between the two techniques [21].

On the other hand, patients with suspected nodal disease, either on CT scan or on PET/CT, should have a pathological confirmation of the lymph node status, either by needle aspiration using endobronchial ultrasonography (EBUS), esophageal ultrasonography (EUS), or by multiple biopsies using navigational bronchoscopy, and/or mediastinoscopy [17]. Aside from those patients with suspicious mediastinal and/or hilar lymph nodes on imaging (cN1-3), those with a centrally located, FDG-non-avid, or large (>3 cm) peripherally located tumor are also advised to undergo invasive mediastinal nodal staging before surgical resection, given that there might be occult lymph node disease.

EBUS provides access to nodal stations 2R/2L, 3P, 4R/4L, 7, 10R/10L, 11–13, and other hilar nodal stations if necessary. On the other hand, EUS provides additional access to stations 3P, 5, 7, 8, and 9, and should be employed if these stations are clinically suspicious. Given that EBUS and EUS aspirations are less invasive than surgical staging (i.e., mediastinoscopy), the use of EBUS and/or EUS as the initial procedure for invasive mediastinal staging is recommended by the European Society of Gastrointestinal Endoscopy, in cooperation with the European Respiratory Society and the European Society of Thoracic Surgeons [22]. Particularly, the combination of EBUS with EUS is preferred over individual tests alone. Nevertheless, in cases where EBUS/EUS does not show evidence of malignant nodal involvement, it is suggested to proceed with surgical staging for potential surgical candidates. 

However, the necessity of confirmatory mediastinoscopy before surgery is being debated due to its limited nodal metastasis detection rate, associated morbidity, and delay in proceeding to definitive surgery [22].

The ASTER trial demonstrated a 79% sensitivity for video-mediastinoscopy to detect nodal metastases compared with 85% for endosonography (combined EBUS/EUS). Confirmatory mediastinoscopy after negative endosonography increased the sensitivity to 94% [23]. On the other hand, the MEDIASTrial aimed to evaluate whether excluding mediastinoscopy would result in an unacceptable increase in unforeseen N2 (uN2) rates during surgery, within a predefined noninferiority limit, considering potential improvements in morbidity, quality of life, and healthcare costs [24]. Eligible patients with proven or suspected resectable NSCLC without distant metastasis and with the usual indications of centrally located FDG-non-avid tumors, large (>3 cm) peripherally located tumors, or cN1-3 positivity on imaging were included. All patients underwent CT and PET imaging, with mandatory endosonographic assessment (systematic EBUS, preferably adding EUS) of nodal station 4R-7-4L. A tumor negative cytology of N2-3 stations was mandatory for all enlarged (short-axis > 10 mm) or FDG-avid (SUV > 2.5) mediastinal nodal stations.

Patients were randomly assigned to undergo immediate lung surgery (tumor resection plus systematic lymph node dissection) or confirmatory mediastinoscopy followed by lung surgery in the absence of nodal metastases. The uN2 rate in the immediate resection group was 8.8%, while it was 7.7% in the mediastinoscopy group, providing a marginal reduction in the uN2 rate by only 1.1% at the cost of a 10-day delay in lung tumor resection, mediastinoscopy-related complications in 6.3% of patients, a mortality rate of 0.6%, and repeat general anesthesia for all involved patients. 

The overall prevalence of N2 disease was 9.9% in the immediate resection group, while it was 16.0% in the mediastinoscopy group [24]. Although this difference in node prevalence between the two groups was not significantly different, it was clinically noticeable. As random assignment involving diagnostic interventions does not affect disease presentation and should lead to similar rates of nodal disease prevalence in each group. Thus, a reason for differences in N2 prevalence could be the lack of blinding in the study because surgeons were not blinded to whether a patient had undergone mediastinoscopy, which could have influenced the thoroughness of nodal evaluation during surgery. However, the number of lymph node stations evaluated intraoperatively was similar between both groups. An adjusted analysis taking into account variations in the baseline characteristics showed comparable results to the primary analysis, suggesting any imbalances to be minimal. Therefore, one potential limitation of the MEDIASTrial is the possibility of chance, leading to uneven group assignment despite randomization [25], meaning that there is still an open debate about the need to perform a mediastinoscopy after negative EBUS/EUS.

Although the need for pathological confirmation of suspected mediastinal lymph nodes has traditionally been reserved to potentially operable patients, this invasive staging might also be useful for patients undergoing definitive chemo-radiotherapy. The recently published SEISMIC trial suggests that systematic endoscopic mediastinal staging in locally advanced NSCLC is more accurate than PET/CT alone in defining the extent of mediastinal involvement. They found a discrepancy in up to 37% of the patients (95% CI, 29–44) in node staging. PET-occult lymph node metastases were identified in 18 (12%) patients including 7% of the patients with occult contralateral N3 diseases. These findings led to clinically significant changes to the radiotherapy treatment plans in all 18 (12%) patients [26], highlighting the utility of this invasive staging. 

## 4. Implications for Treatment

### 4.1. Surgical Resectability

The role of surgery in treating patients with stage III tumors, particularly those with mediastinal nodal involvement (N2), has been a topic of discussion for many years. This debate is largely due to varying inclusion criteria in clinical trials and a lack of precise definitions such as single-station N2, multiple-station N2, and bulky N2, among others. 

Two important randomized trials have evaluated the role of surgery in this population. The one from Albain et al. tested concurrent chemo-radiotherapy followed by resection versus standard concurrent chemotherapy and definitive radiotherapy (without resection) in patients with T1-3pN2 tumors [27]. The EORTC trial from Meerbeeck et al. compared resection versus radiotherapy, both after induction chemotherapy, in patients with stage IIIA-N2 [26]. Neither study showed an overall survival (OS) benefit with the use of surgery, which was associated with higher morbidity and mortality. However, given the heterogeneity of this population, these trials did not sufficiently evaluate the nuances presented by N2 disease and the possible oncological benefit of surgery in specific clinical situations [17]. 

Therefore, in order to determine the role of surgery in resectable stage III tumors, a clear and consistent definition of resectable disease is essential. This would allow for a better comparison and interpretation of research findings and the development of optimal treatment recommendations for each subgroup of stage III NSCLC. 

With that in mind, the EORTC Lung Cancer Group, along with other international societies, has proposed a consensus definition of stage III NSCLC resectability to be used in clinical trials. This definition assumed that patients were medically operable, that all types of major pulmonary resection could be considered, and that resectability had to be assessed upfront before any treatment. This comprehensive project included a systematic review [28], a survey [29], and clinical case discussions [30].

This consensus was established in 2023, based on the input from thoracic surgeons, radiation oncologists, medical oncologists and/or pneumo-oncologists, pulmonologists, radiologists, and pathologists. One important point was that pathological confirmation of N2 was deemed mandatory, except for bulky and invasive N2 where the multidisciplinary tumor board decides that the risks outweigh the benefits and if confirmation does not influence the treatment strategy. 

In this consensus definition, N2 disease was divided into “single-station” (non-bulky, non-invasive), “multiple-station” (non-bulky, non-invasive), “bulky”, and “invasive”. Single-station N2 was considered resectable, while for multiple-station N2, a case-by-case discussion should be conducted, where highly selected patients with “limited” N2 multi-station involvement may be included in clinical trials involving surgery. However, the exact number of nodes/stations defining “limited” cannot be defined. On the other hand, most tumors with “bulky” N2 disease are considered unresectable, as are all tumors with “invasive” N2 [29,31]. 

### 4.2. Peri-Operative Treatment

The optimal treatment for patients with potentially resectable N2 NSCLC is unclear due to the heterogeneity of the disease, a lack of a universal definition of resectability in previous trials, and comparable outcomes with different treatment approaches. Nonetheless, it is consensually agreed that patients with tumors with N2 involvement should receive some form of systemic treatment (chemotherapy, immunotherapy, and/or targeted therapy) given their high risk of relapse [4]. Systemic treatment is usually given before surgery, but it is also possible to perform upfront surgery for tumors with single-station N2 involvement, followed by adjuvant treatment.

#### 4.2.1. Neoadjuvant Systemic Treatment

Neoadjuvant chemotherapy is the preferred approach [17], as adding radiotherapy to induction chemotherapy for patients with operable stage IIIA-N2 disease did not improve the outcomes compared to induction chemotherapy alone [32].

Neoadjuvant treatment with chemotherapy has been evaluated in multiple clinical trials (Table 1). Song et al. published a metanalysis of 13 clinical trials that evaluated the role of preoperative chemotherapy followed by surgery versus surgery alone, with a 5% absolute benefit on 5-year overall survival (OS) benefit (hazard ratio (HR) 0.84, 95% CI, 0.77–0.92) [33]. In the post hoc analysis, the subgroup of patients with stage III NSCLC had a similar HR (HR 0.84, 95% CI, 0.75–0.95), but there was no detailed information for patients with N2 involvement (Table 2). These results are similar to those reported in other studies and meta-analyses, which infers that the benefit from neoadjuvant chemotherapy is similar to that attained with adjuvant chemotherapy [34]. 

Nonetheless, in the hope of improving survival outcomes, a new strategy of neoadjuvant treatment has been evaluated with the combination of chemotherapy plus immunotherapy. 

The phase III CheckMate 816 trial randomly assigned 358 patients with stage IB to IIIA resectable NSCLC without EGFR/ALK oncogene addictions to receive nivolumab plus platinum-based chemotherapy or platinum-based chemotherapy alone, followed by resection. Around 64% of patients presented with stage IIIA, however, there was no further specific information on what type of N components were included in this population. The pathological complete response (pCR) for stage IIIA was 24% (95% CI, 18–31) in the combination group and 2.2% (95% CI, 0.6–5.6) in the chemotherapy alone group. The event-free survival (EFS) for the stage IIIA subgroup undergoing chemo-immunotherapy was almost twice as long compared to the chemotherapy alone group (31.6 versus 15.7 months, respectively; HR 0.54, 95% CI, 0.37–0.80). Overall, the surgical outcomes were also better in the combination group. For patients with stage IIIA disease, there were more patients receiving surgical treatment (83.2% versus 72.2%), a lower rate of disease progression before surgery (8% versus 13.9%), and a higher complete resection (R0) rate (83% versus 78.3%) [35].

The phase II TD-FOREKNOW study also combined chemotherapy with an anti-PD-1 drug (camrelizumab), showing a benefit of this regimen compared to chemotherapy alone in patients with stage III NSCLC [36]. Unfortunately, no specific data on N2 tumors are available.

Given the limited benefit of immunotherapy in patients with EGFR/ALK oncogene-addicted NSCLC, there are trials being specifically developed for this population (Table 1). Two phase II single-arm trials have evaluated the benefit of neoadjuvant treatment with osimertinib for patients with EGFR-mutant NSCLC. The first one, from Aredo et al., enrolled 27 patients in the United States [37]; the second one, the NEOS trial, enrolled 38 patients in China [38]. Stage III represented 33.3% of the patients of the former and 65% of the latter. In the first trial, major pathological response (MPR) was achieved in 15% of the patients, but none attained a pCR. In the NEOS trial, MPR was achieved in 11% and pCR in 4% of the patients. Again, outcome information on patients with N2 disease was not provided [37,38]. Results of the NeoADAURA study are now eagerly awaited, which is randomizing patients to osimertinib with or without chemotherapy versus chemotherapy alone in patients with resectable stage II-IIIB N2 EGFR mutation-positive NSCLC, followed by surgery and adjuvant treatment (as per the investigator’s choice, which could include adjuvant osimertinib) [39]. 

**Table 1:** Clinical trials design and patient characteristics.

Type of Treatment	Trial Name	Phase	Treatment Arms	Adenocarcinoma/ Non-Squamous	TNM Version	N of Patients	Stage III	N2 Patients	N2 Single -Station	N2 Multi-Station	Pre-Treatment Nodal Pathological Confirmation Required	Technique for Pathological Confirmation (Pre-Treatment)
**Neoadjuvant Setting**												
**CT**	Song et al. [33]	MA	CT	NA	3–6	1637	50%	NA	NA	NA	NA	NA
			BSC	NA		1587	48%	NA	NA	NA		
**CT+ Immunotherapy**	Checkmate-816 [35]	III	CT + Nivolumab	51%	8	179	63%	NA	NA	NA	Yes	Mediastinoscopy, thoracotomy, or EBUS
			CT	47%		179	64%	NA	NA	NA		
	TD-FOREKNOW [36]	II	CT + Camrelizumab	37%	8	43	100%	79%	NA	NA	No	NA
			CT	29%		45	100%	69%	NA	NA		
**Targeted therapy**	Aredo et al. [37]	II	Osimertinib	100%	7	27	33%	NA	NA	NA	No	NA
	NEOS [38]	IIB	Osimertinib	100%	8	38	65%	17%	NA	NA	Yes, if PET-CT was negative	EBUS
**Adjuvant Setting**												
**CT**	LACE [34]	MA	CT	51%	5–6	2281	27%	23%	NA	NA	--	--
			Placebo	51%		2303	27%	22%	NA	NA		
**CT+ Immunotherapy**	PEARLS/KEYNOTE 091 [40]	III	Pembrolizumab	67%	7	590	30%	21%	NA	NA	--	--
			Placebo	62%		587	28%	18%	NA	NA		
	IMpower 010 [41,42]	III	Atezolizumab	65%	7	507	40%	30%	NA	NA	--	--
			BSC	67%		498	42%	30%	NA	NA		
**Targeted therapy**	CTONG1104 [43]	III	Gefitinib	92%		111	65%	64%	NA	NA	--	--
			CT	95%		111	64%	65%	NA	NA	--	--
	ADAURA [44,45]	III	Osimertinib	96%	7	339	35%	31%	NA	NA	--	--
			Placebo	97%		343	34%	30%	NA	NA		
	ALINA [46]	III	Alectinib	95%	7	130	53%	49%	NA	NA	--	--
			CT	98%		127	55%	52%	NA	NA		
**Perioperative Setting**												
**CT+ Immunotherapy**	AEGEAN [5]	III	CT + Durvalumab	54%	8	366	71%	50%	39%	9%	Yes, if PET-CT was negative	Mediastinoscopy, thoracotomy, or EBUS
			CT + Placebo	48%		374	70%	50%	35%	11%		
	KEYNOTE 671 [6]	III	CT + Pembrolizumab	57%	8	397	70%	42%	NA	NA	Yes, if PET-CT was negative	Mediastinoscopy, thoracotomy, or EBUS
			CT + Placebo	57%		400	70%	47%	NA	NA		
	Checkmate 77T [7]	III	CT + Nivolumab	49%	8	229	65%	65%	26%	14%	NA	NA
			CT + Placebo	49%		232	64%	64%	23%	16%		
	Neotorch [8]	III	CT + Toripalimab	22%	8	202	100%	68%	NA	NA	Yes, if PET-CT was negative	Mediastinoscopy, thoracotomy, or EBUS
			CT + Placebo	22%		202	99%	72%	NA	NA		
	Rationale 315 [9]	III	CT + Tislelizumab	NA	8	226	58%	36%	NA	NA	NA	NA
			CT + Placebo	NA		227	59%	35%	NA	NA		
	NADIM II [47]	II	CT + Nivolumab	44%	8	57	100%	72%	33%	39%	Yes, if N2 disease was suspected by PET-CT	Mediastinoscopy, thoracotomy, or EBUS
			CT	38%		29	100%	55%	17%	38%		

BSC: best standard of care; CT: chemotherapy; EBUS: endobronchial ultrasound; NA: not available.

**Table 2:** Clinical trials in the neoadjuvant and adjuvant setting—outcomes.

Trial Name	Treatment Arms	pCR Rate—% (95% CI) *		EFS/DFS/PFS—Median (95% CI) & HR (95% CI) *			OS—Median (95% CI) & HR (95% CI) *		Surgical Outcomes	
		All Patients	N2/Stage III	End-Point	All Patients	N2/Stage III	All Patients	N2/Stage III	R0 §	Did Not Complete Surgery
**NEOADJUVANT**										
**Song et al. [33]**	CT	NA	NA	NA	NA	NA	NA. HR 0.84 (0.77–0.92)	**Stage III**: NA. HR 0.84 (0.75–0.95)	NA	NA
	Placebo									
**Checkmate 816 [35]**	CT + Nivolumab	24% (18.0–31.0)	**Stage IIIA**: 23% (15.6–31.9)	EFS	31.6 (30.2-NR) vs. 20.8 (14.0–26.7) HR 0.63 (0.43–0.91)	**Stage IIIA**: 31.6 (26.6–NR) vs. 15.7 (10.8–22.7) HR 0.54 (0.37–0.80)	NR vs. NR HR 0.62 (0.36–1.05)	NA	83%	16%
	CT	2% (0.6–5.6)	**Stage IIIA:** 0.9% (<0.1–4.7)						78%	21%
**TD-FOREKNOW [36]**	CT + Camrelizumab	32.6% (19.1–48.5)	NA	EFS	NR vs. NR HR 0.52 (0.21–1.29)	NA	NA	NA	NA	NA
	CT	8.9% (2.5–21.2)								
**Aredo et al. [37]**	Osimertinib	0%	NA	DFS	32.4 (25.9-NR)	NA	NA	NA	89%	NA
**NEOS [38]**	Osimertinib	4%	NA	NA	NA	NA	NA	NA	94%	NA
**ADJUVANT**										
**LACE [34]**	CT	--	--	DFS	NA HR 0.84 (0.78–0.91)	NA	NA HR 0.89 (0.82–0.96)	**Stage III**: NA HR 0.83 (0.72–0.94)	--	--
	Placebo									
**PEARLS/KEYNOTE 091 [40]**	Pembro-lizumab	--	--	DFS	53.6 (39.2-NR) vs. 42.0 (31.3-NR) HR 0.76 (0.63–0.91)	**Stage IIIA**: NA HR 0.92 (0.69–1.24)	NR vs. NR HR 0.87, 95% CI, 0.67–1.15	NA	--	--
	Placebo	--	--						--	--
**IMpower 010 [41,42]**	Atezolizumab	--	--	DFS	**Final DFS analysis:** ITT: 65.6 vs. 47.8, HR 0.85 (0.71–1.01) PD-L1 ≥ 50%: NR vs. 41.1, HR 0.48 (0.32–0.72) PD-L1 1–49%: 68.5 vs. 37.3, HR 0.70 (0.55–0.91)	**Primary DFS analysis:** **All N2 & PD-L1 ≥ 1%:** 32.3 (24.2–NE) vs. 21.3 (15.7–31.4) HR 0.66 (0.44–0.99) **All N2:** 30.2 (24.0–42.3) vs. 24.1 (18.0–31.4) HR 0.83 (0.61–1.13)	**Second OS analysis:** **II-IIIA and:** PD-L1 ≥ 50%: NE vs. 87.1, HR 0.47 (0.28–0.77) PD-L1 1-49%: NE vs. 87.1, HR 0.77 (0.56–1.06) PD-L1 < 1%: NA	**Primary OS analysis:** **All N2 and:** PD-L1 ≥ 50%: NE vs. NE. HR 0.36 (0.14-0.95) PD-L1 1–49%: NE vs. NE. HR 1.38 (0.73–2.61) PD-L1 < 1%: NE vs. NE. HR 1.26 (0.72–2.22)	--	--
	BSC	--	--						--	--
**CTONG1104 [43]**	Gefitinib	--	--	DFS	30.8 (26.7–36.6) vs. 19.8 (15.4–23.0) HR 0.56 (0.40–0.79)	**All N2**: NA. HR 0.52 (0.34–0.80)	75.5 (46.6-NE) vs. 62.8 (45.8-NE) HR 0.92 (0.62–1.36)	**All N2**: NA. HR 0.92 (0.58–1.45)	--	--
	CT	--	--						--	--
**ADAURA [44,45]**	Osimertinib	--	--	DFS	NR (38.8-NE) vs. 27.5 (22–35) HR 0.20 (0.14-0.30)	**Stage IIIA**: NA. HR 0.12 (0.07–0.20)	At 5 years: 88% (83–91%) vs. 78% (73–82%) HR 0.49 (0.34–070)	**Stage IIIA**: At 5 years: 85% (76–91%) vs. 67% (57–75%) HR 0.37 (0.20–0.64)	--	--
	Placebo	--	--						--	--
**ALINA [46]**	Alectinib	--	--	DFS	NR vs. 41.3 (28.5-NC) HR 0.24 (0.13–0.45)	**All N2**: NA. HR 0.21 (0.09–0.47)	NA	NA	--	--
	CT	--	--						--	--

BSC: best supportive care; CI: confidence interval; DFS: disease-free survival; EFS: event-free survival; HR: hazard ratio; ITT: intention to treat; NA: not available; NE: Not estimable; NR: not reached; ORR: objective response rate; OS: overall survival; pCR: pathological complete response; PFS: progression-free survival. * We preferentially presented the data on N2 outcomes—if these were not available, then we presented the data on stage III outcomes (as it comprises patients with tumors with N2 involvement). § R0: complete surgical resection (i.e., the surgical margin is microscopically-negative for residual tumor).

#### 4.2.2. Adjuvant Systemic Treatment

As previously mentioned, patients with resectable, proven N2 disease usually receive induction treatment before surgery. However, there are tumors considered to be cN0 or cN1 at diagnosis that turn out to have pN2 involvement upon resection (“unforeseen N2 disease”). In these cases, adjuvant treatment should be provided after surgery. The LACE pooled analysis revealed that there was a 5-year OS absolute improvement of 5.4% from adjuvant chemotherapy (HR 0.89, 95% CI 0.82–0.96) in the overall population, with a similar benefit in patients with stage III disease [33].

Given the modest benefit provided by chemotherapy, adjuvant treatment with immunotherapy has been evaluated in two phase III trials as an additional 1-year treatment after chemotherapy. The Keynote-091/PEARLS trial compared pembrolizumab versus placebo, and the IMpower010 trial tested atezolizumab vs. the best standard of care (BSC). There was a statistically significant benefit in disease-free survival (DFS) in both trials favoring immunotherapy (HR 0.76 and 0.81 (first analysis), respectively) [40,41]. However, the IMpower010 5-year DFS analysis demonstrated that this benefit was only significant for the stage II-IIIA PD-L1 positive tumors (HR 0.70, 95% CI 0.55–0.91) [42].

About 19.5% of the patients in the Keynote-091/PEARLS trial had N2 disease with a non-significant benefit in DFS in this subgroup with an HR of 0.92 (95% CI 0.69–1.24). On the other hand, there was an 11-month absolute DFS benefit (HR 0.66, 95% CI 0.44–0.99) among patients with N2 involvement and positive PD-L1 in the IMpower010 study. In the 5-year second OS analysis for the IMpower010, a benefit was seen in the high PD-L1 expressing tumors (HR 0.47, 95% CI 0.28–0.77). In a subgroup analysis, there was a significant benefit on OS for patients with PD-L1-high N2 tumors (HR 0.36, 95% CI, 0.14–0.95), but not for N0 or N1. However, no updated data for DFS nor OS are available for this subgroup and OS data are not yet mature in the Keynote-091 [40,41,42].

Targeted therapies have also been evaluated in the adjuvant setting. In patients with EGFR mutated tumors, trials testing adjuvant treatment with first-generation tyrosine kinase inhibitors (TKIs) such as geftinib showed an improvement in DFS, but not in OS [43].

Nonetheless, the more recent ADAURA study compared the third-generation TKI osimertinib for 3 years against the placebo, after an optional adjuvant chemotherapy. Around 30% of patients had N2 tumors and 60% of patients received adjuvant chemotherapy. There was a significant 37% difference in DFS favoring osimertinib in the overall population (HR 0.20, 99% CI, 0.14–0.30), which was greater in patients with IIIA disease—HR 0.12 (95% CI, 0.07–0.20) [44]. Similarly, there was a significant 5-year OS benefit in the overall population (88% vs. 78%, HR 0.49, 95% CI, 0.34–0.70), which was greater in patients with stage IIIA disease (85% vs. 67%, HR 0.37, 0.20–0.64), but not significant for patients with disease stage IB and II [45]. Unfortunately, there are no details regarding this benefit specifically for patients with N2 disease.

Finally, for patients with resected, stage IB-IIIA, ALK-positive NSCLC, the recommended treatment after surgery is still adjuvant platinum-based chemotherapy. Recently, however, the ALINA trial compared four cycles of platinum-based adjuvant chemotherapy against 2 years of alectinib. An overall benefit in DFS was observed in the alectinib group including in patients with stage IIIA (HR 0.25, 95% CI 0.12–0.53). At the moment of this analysis, OS data are still immature [46].

#### 4.2.3. Perioperative Systemic Treatment

Although the benefit of neoadjuvant treatment with chemotherapy or chemo-immunotherapy is clear, there is still the open question of whether continuing this treatment after surgery could lead to a more effective elimination of micrometastatic disease and, thus, increase the likelihood of cure. With this perioperative strategy in mind, one phase II and five phase III trials testing different anti-PD-(L)1 antibodies will be discussed [5,6,7,8,9,47] (Table 3).

All trials combined platinum-based chemotherapy with immunotherapy as neoadjuvant treatment, and only the Neotorch trial included one cycle of postoperative chemotherapy in addition to immunotherapy. Postoperative treatment duration was 6 months in the NADIM II trial, but around 1 year in the other studies. Frequency varied among trials, with KEYNOTE-671 and Neotorch trials administering immunotherapy every 3 weeks for a total of 13 cycles, the Checkmate 77T and AEGEAN trials every 4 weeks for 12 cycles, and RATIONALE-315 every 6 weeks for approximately eight cycles. Pathological assessment of N2 disease was mandatory in the NADIM II trial, while it was optional (i.e., according to each site’s practice) in the other studies. 

**Figure 2:**
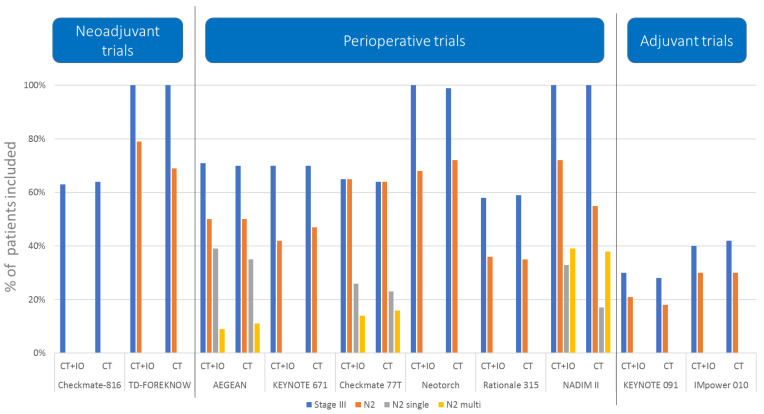
Proportion of patients with stage III, N2 all, N2 single, and N2 multiple disease in the neoadjuvant, perioperative, and adjuvant trials comparing chemotherapy (CT) versus chemo-immunotherapy (CT-IO).

The proportion of N2 disease was considerably high in these perioperative trials if compared to the purely adjuvant trials, with a prevalence going from 35% to 72%. Most of these trials included more than 50% of patients with N2 disease (Figure 2). Interestingly, the proportion of multiple-station N2 disease was very high in the phase II NADIM II trial (39%), and were modest in the AEGEAN (10%) and Checkmate 77T (15%) trials. The other studies did not specify the proportion of multiple-station N2 tumors enrolled.

All trials showed an increased MPR and pCR rate with the addition of immunotherapy. Among the 17 patients with N2 disease in the NADIM II trial, the pCR rate was 39% in the chemo-nivolumab arm compared to only 6.2% in the chemotherapy arm. Interestingly, the pCR rate was similar in both patients with single-station N2 (42.1%) and multiple-station N2 (36.4%) [47]. In the AEGEN trial, a numerically higher pCR rate was reported within the N2 single-station group compared to the multi-station group receiving immunotherapy (18.4% vs. 8.8%, respectively), but this was much lower than what was reported in the NADIM II trial.

#### 4.2.4. Perioperative Chemo-Immunotherapy—Pooled Analysis for N2 Disease

A recently published systematic review and meta-analysis by Banna et al. included trials testing neoadjuvant immunotherapy-chemotherapy vs. neoadjuvant chemotherapy alone, with, or without the addition of adjuvant immunotherapy, among patients with resectable NSCLC [49]. The authors searched EMBASE, PubMed, the Cochrane Central Register of Controlled Trials, and the Cochrane Database of Systematic Reviews through 1 November 2023, and in oncology conference proceedings from 1 January 2008 to 1 November 2023. They included eight trials: two that tested chemo-immunotherapy as a neoadjuvant treatment only (CheckMate 816 and TD-FOREKNOW trials [35,36]) and six that assessed chemo-immunotherapy as a perioperative strategy (AEGEAN, KEYNOTE 671, Checkmate 77T, Neotorch, Rationale 315 and NADIM II [5,6,7,8,9,47]). Combined immunotherapy demonstrated a statistically significant EFS benefit across all trials for the overall population, being generally larger in patients with stage III tumors. 

**Figure 3:**
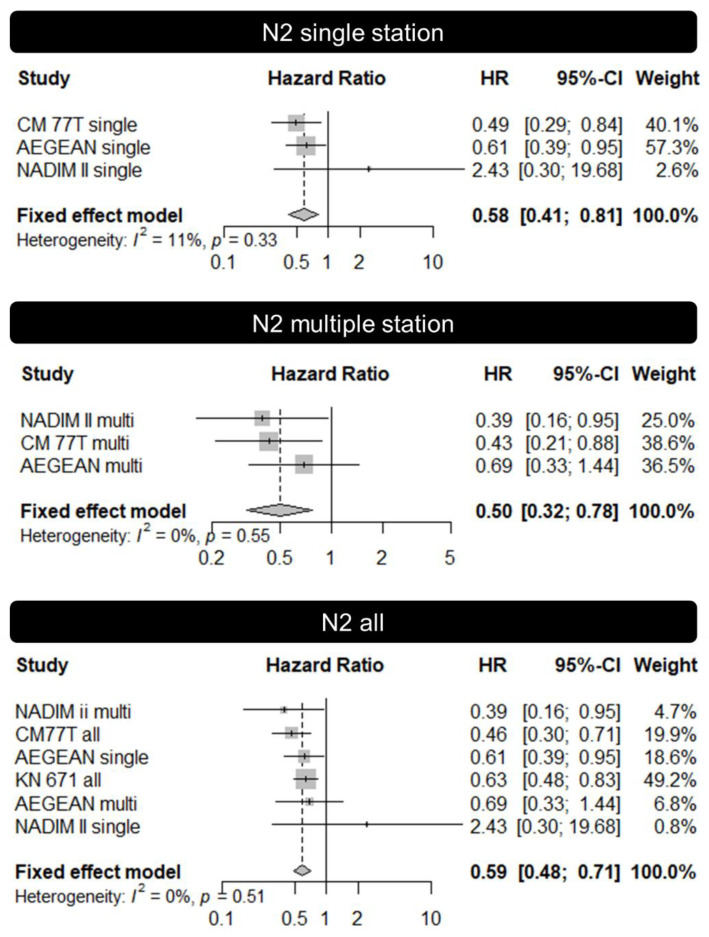
Meta-analysis of event-free survival for immuno-chemotherapy compared to chemotherapy alone, according to the N2 status (N2 single-station, N2 multiple-station, and all N2).

Taking these results into account, we searched the EFS benefit specifically for N2 disease in these trials, something that had not been explored in the systematic review by Banna et al. [49]. An analysis of EFS for the subgroup of patients with N2 tumors was reported in the AEGEAN, Checkmate 77T, KEYNOTE 671, and NADIM II trials, with the HR usually being similar or better in the N2 subgroup compared to the overall population (Table 3). In order to assess the impact of chemo-immunotherapy on EFS, we performed a new meta-analysis based on the above-mentioned trials, which were identified in the systematic review by Banna et al. For this, we estimated the pooled hazard ratios (pHR), using chemotherapy as the reference group. Individual point estimates were extracted from the publications together with the 95% confidence intervals (overall N2 and in the two subgroups considered—N2 single-station and N2 multi-station). Variance of the point estimate on the log scale was obtained from the 95% confidence interval. pHR were obtained using fixed effects modeling. Given that no significant heterogeneity was detected, it was not necessary to perform a random effects model. As demonstrated in Figure 3, the pHR for N2-single station tumors was 0.58, which was close to the one of N2-multi station disease (pHR 0.59), meaning that the magnitude of benefit from the addition of immunotherapy seems to be similar in both subgroups of patients.

OS data for the overall population has been reported for the KEYNOTE-671 and Neotorch trials with a statistically significant benefit favoring combined immunotherapy preoperative treatment. Importantly, in the KEYNOTE 671 trial, the OS benefit was similar in the N2 subpopulation compared to the overall population (HR of 0.74 versus HR of 0.72, respectively).

While most of these trials did not provide specific information on the assessment of mediastinal nodal involvement and N2 subtypes, they laid the foundation for integrating immunotherapy into the perioperative strategy for patients with resectable NSCLC with N2 involvement including in multiple-station N2.

#### 4.2.5. Postoperative Radiotherapy (PORT)

After multiple retrospective series suggesting that PORT after complete tumor resection for patients with N2 NSCLC could be beneficial [50], this was prospectively evaluated in the LungART randomized trial [51]. This study explored the use of modern mediastinal PORT in patients with completely resected NSCLC (R0) with histo/cytologically proven N2 involvement. However, after surgical committee review, 41% of cases were reclassified as having uncertain resection (R uncertain) and 30% as incomplete resection for nodal extracapsular extension (R1). A total of 501 patients were randomized to PORT (54 Gy in 27–30 fractions, on five consecutive days per week) or observation. 

Although there was less locoregional relapse in the PORT arm, the 3-year DFS was 47% (95% CI, 40–54) with PORT versus 44% (95% CI, 37–51) without PORT, and the median DFS was 30.5 months (95% CI, 24–49) in the PORT versus 22.8 months (95% CI, 17–37) in the control group (HR 0.86, 95% CI 0.68–1.08). Even though the data were immature, there was no difference in the 3-year OS, with 67% in the PORT group versus 69% in the control group—adjusted HR 0.97 (95% CI 0.73–1.28). Late grade 3–4 cardiopulmonary toxicity was reported in 26 patients (11%) in the PORT group versus 12 (5%) in the control group. Therefore, PORT cannot be recommended as a standard treatment for patients with completely resected (R0) stage III N2 NSCLC [51]. 

However, careful attention needs to be given to the quality of surgical resection (i.e., the assessment of the status of extracapsular invasion as well as the degree of certainty of resection). Therefore, patients who are at a higher rate of local recurrence (such as those with R1 or R2 resection) might still benefit from PORT.

### 4.3. Radical Treatment in Unresectable Locally Advanced NSCLC

Patients diagnosed with unresectable locally advanced NSCLC typically undergo a combined treatment approach involving platinum-based chemotherapy and radiotherapy as a definitive treatment [4]. A significant advancement in this setting was the phase III PACIFIC trial, in which patients were randomized to receive 1 year of either durvalumab or the placebo as a consolidation treatment for patients without disease progression following completion of the standard concomitant treatment with chemo-radiotherapy. Around half of the patients presented with stage IIIA, and 45% with stage IIIB [52]. Updated data for the overall population reported a sustained benefit in progression-free survival (PFS; 16.9 versus 5.6 months—HR 0.55, 95% CI, 0.45–0.68) and on OS (47.5 versus 29.1 months—HR 0.72, 95% CI, 0.59–0.89), favoring the durvalumab group [53]. Prospective real-world evidence from the PACIFIC-R study, which included a large cohort of nearly 1400 participants, further confirmed these findings [54]. 

However, in some situations, stage IIIA-N2 tumors may be deemed resectable, but the relative benefit of surgery is unclear, as mentioned before. A post hoc exploratory analysis of clinical outcomes in patients with or without stage IIIA-N2 NSCLC in the PACIFIC trial was performed with data from the primary analysis data cutoff; 40.3% of the patients had stage IIIA-N2 disease, while the subgroup without stage IIIA-N2 disease included all other stages (IIIA-nonN2 and stage IIIB). Patients with stage IIIA-N2 disease had a benefit in PFS (HR 0.46, 95% CI, 0.33–0.65) and in OS (HR 0.56, 95% CI, 0.39–0.79) with the addition of durvalumab [54]. 

Still, the question regarding the optimal local treatment for stage IIIA-N2 tumors (surgery or radiotherapy) remains open. Thus, future research should aim to evaluate the effects of the PACIFIC regimen on progression patterns in patients with stage IIIA-N2 disease. These data will be valuable in guiding discussions about the most effective multimodal treatment strategy for patients, particularly considering the potential benefits of surgery for improving locoregional control. It is important to note that the above-mentioned analysis did not include an assessment of locoregional failure, as radiotherapy planning parameters were not consistently collected according to the study protocol. This limitation hindered the ability to establish correlations between disease progression and previous radiation treatments in all cases.

Although the use of adjuvant durvalumab led to a remarkable improvement in OS [55], only 33.1% of patients included in the PACIFIC trial were alive 5 years after diagnosis. Thus, the PACIFIC-2 trial was designed to evaluate whether giving durvalumab concurrently with platinum-based chemoradiotherapy, followed by consolidation durvalumab, would improve the survival in patients with stage III NSCLC. A total of 328 patients were included, of which 56% presented with N2 disease. Surprisingly, the earlier administration of durvalumab did not lead to improved PFS or OS in the overall population [56]. Again, no specific information regarding patients with N2 disease has been provided thus far.

Therefore, instead of anticipating the use of immunotherapy, another strategy would be to combine durvalumab with other immune checkpoint inhibitors. With that in mind, the phase II COAST trial evaluated the use of durvalumab alone or combined with the anti-CD73 monoclonal antibody oleclumab or anti-NKG2A monoclonal antibody monalizumab as consolidation therapy after chemo-radiotherapy. There was a benefit in ORR and PFS in both combinations in comparison to durvalumab alone, however, specific data for patients with N2 disease are not available [48]. The efficacy of these combined immunomodulatory regimens is being further investigated in the PACIFIC-9 phase 3 randomized trial (NCT05221840). 

For patients with EGFR-mutated tumors, the LAURA trial tested the efficacy and safety of osimertinib as maintenance therapy in patients with locally advanced, unresectable, stage III NSCLC without disease progression following definitive chemoradiation therapy. Median PFS was dramatically increased, going from 5.6 months with the placebo to 39.1 months with osimertinib (HR 0.16, 95% CI, 0.10–0.24). Data from the interim analysis of overall survival are still immature, but it reported a 36-month OS of 84% among patients treated with osimertinib vs. 74% among those treated with the placebo (HR 0.81, 95% CI 0.42–1.56) [57]. Nonetheless, there are no specific data on patients with N2 tumors. 

## 5. Areas of Future Research

In the first part of this review, we highlighted the heterogeneity of N2 disease and the great advancements made in the last decade in terms of its diagnosis and treatment. When looking at the recent neoadjuvant, perioperative, and adjuvant trials, it is clear that patients with stage III-N2 NSCLC present the largest benefit from the use of systemic therapy compared to patients with earlier disease stages (Table 2 and Table 3). This is certainly because most patients with N2 disease likely have micrometastatic disease at the time of diagnosis, and therefore, local therapy is not enough to cure them. Nonetheless, there are still a myriad of open questions regarding the choice of treatment and its optimization for this subgroup of patients.

### 5.1. Best Local Treatment Modality for Operable Patients with Resectable N2 Disease

After decades of research, it is still not clear what the best local treatment for operable patients with resectable N2 disease is: surgery or radiotherapy? For years, they have been considered as equivalent. However, after the publication of the PACIFIC trial [58], there was a “shift” in clinical practice favoring chemo-radiotherapy, as these patients could afterward benefit from adjuvant immunotherapy. Conversely, in the last couple of years, the publication of multiple immunotherapy trials in the peri-operative space [5,6,7,8,9] brought about another “shift”, but this time in favor of surgery.

Although a head-to-head clinical trial comparing induction chemo-immunotherapy followed by surgery versus chemo-radiotherapy followed by adjuvant durvalumab could theoretically be proposed, it would be very hard to have true equipoise in the different treatment modalities. Moreover, enrollment would also be difficult in such a trial, given that both physicians and patients may have strong preferences toward one treatment or another, and it would not be possible to perform blinding. Finally, funding of such a large academic trial would also be a challenge.

Therefore, the use of real-world data could be used to try to tackle this question. However, one of the problems of using retrospective real-world data is the absence of a systematic collection of important variables when assessing the patient’s operability such as functional assessment (e.g., lung function tests, cardiac exams) or the tumor’s resectability—the complete imaging work-up (i.e., contrast-enhanced CT scan, [18F] FDG PET/CT, and brain imaging) and the invasive mediastinal staging for suspected N2 disease. Moreover, data on the completeness of surgery or on the radiotherapy planning are frequently difficult to obtain, and the follow-up is not standardized. Thus, the EORTC Lung Cancer Group is about to launch a prospective stage III platform, which is to be part of the larger SPECTA platform (NCT02834884) [59]. Patients with stage III NSCLC will be prospectively enrolled, and a complete assessment at the baseline and at specified timepoints will be performed. This will include a vast translational research component as well as the quality of life and patients’ preferences. Hence, this database could potentially be used to compare the outcomes (i.e., survival, quality-of-life and others) of patients who receive multimodality treatment including surgery compared to those benefiting from chemo-radiotherapy. It will also be useful to assess these outcomes according to a more granular definition of N2 disease (i.e., single-station N2 vs. multiple-station vs. N2-bulky and invasive). Furthermore, within the “multiple-station N2” category, it would be interesting to assess how many stations are affected, at which levels and zones, and how this correlates with outcomes.

### 5.2. “Borderline” Resectable Tumors

Another important question regards the best multimodality regimen for patients with “borderline” resectable N2 tumors, which could benefit from a neoadjuvant treatment in order to downstage the tumor and lymph nodes and render it resectable. This strategy was frequently employed in the chemotherapy-only era as it had been demonstrated that patients with a downstaging of cN2 to ypN0 disease had a more favorable long-term outcome [60]. 

Nonetheless, now that immunotherapy is also available in the neoadjuvant setting, there might be a tendency to mirror this approach and use chemo-immunotherapy as a strategy to render resectable a “borderline resectable” N2 tumor. However, all the above-mentioned trials in the neoadjuvant/perioperative setting only included patients with N2 disease considered to be upfront resectable (i.e., regardless of the use of systemic therapy).

Therefore, patients with “borderline resectable” tumors for whom surgery is being considered should be included in clinical trials. One possibility is the phase II MDT-BRIDGE clinical trial, which evaluates, in initially deemed resectable/borderline resectable stage IIB-IIIB (N2) NSCLC, the use of neoadjuvant durvalumab plus the investigator’s choice of platinum-based chemotherapy followed by multidisciplinary tumor board assessment of resectability. Patients with tumors deemed resectable will receive a further 1–2 cycles of combined neoadjuvant chemo-immunotherapy followed by surgery; patients with tumors deemed unresectable will receive chemo-radiotherapy. After surgery/chemo-radiotherapy, all patients will receive maintenance durvalumab for up to 1 year, as per the CheckMate77T and PACIFIC regimens, respectively [61]. 

### 5.3. Tailored Treatment

#### 5.3.1. Actionable Genomic Alterations

Another open question is which is the best systemic treatment in the early setting for patients with actionable genomic alterations and N2 involvement. For ALK-positive tumors, the phase II ALNEO trial (NCT05015010) will investigate the role of alectinib in potentially resectable locally advanced ALK-positive NSCLC. Patients with stage III ALK-positive NSCLC (any T with N2, T4N0-1) will receive neoadjuvant alectinib 600 mg for two months. In the absence of disease progression, patients will undergo surgery within 3 weeks. After surgery, they will complete their treatment with 24 cycles (2 years) of adjuvant alectinib [62].

Furthermore, the phase II NAUTIKA 1 clinical trial (NCT04302025) is evaluating the use of multiple neoadjuvant and adjuvant therapies in biomarker-selected patients with resectable stage IB-III NSCLC including N2 disease [63]. Molecular testing will be performed to detect one of the following abnormalities and treatments: ALK fusion (alectinib), ROS1 fusion (entrectinib), NTRK1/2/3 fusion (entrectinib), BRAF V600 mutation (vemurafenib plus cobimetinib), RET fusion (pralsetinib), PD-L1 (atezolizumab, and during the first cycle, patients will also receive low-dose SBRT (8Gy × 3)), and KRAS G12C (divarasib) as determined by an FDA-approved test [62]. This is a smart trial design, as it will boost enrolment for rare genomic alterations such as ROS1/RET/NTRK fusions, as all patients benefit from a complete molecular screening at diagnosis. Moreover, it will allow us to understand the value of these targeted therapies in the early-stage setting, especially for patients with tumors at a high-risk of recurrence such as those with N2 involvement.

#### 5.3.2. Genomic Signatures

Although the added value of immunotherapy in the early-stage setting is now undeniable, there is still a lot of uncertainty regarding which patients will benefit most. The Besides PD-L1 positivity and higher stage at diagnosis (including N2 disease), there are no other robust markers so far that are ready to be used in the clinic.

In order to shed light on this question, a 4-gene inflammatory signature comprised of CD8A, STAT1, LAG3, and CD274 (encoding PD-L1) was assessed by the RNA-sequencing of the baseline tumor samples of patients included in the CheckMate-816 study. In the chemo-immunotherapy group, patients who achieved MPR and pCR had a higher 4-gene inflammatory signature score than those who did not. This was not observed in the control arm, which could be due to the limited number of pathological responses. A high 4-gene inflammatory gene score was associated with a numerically higher EFS in comparison to a low score (HR 0.65, 95% CI 0.30–1.39), but this was non-significant. These results provide the base for future studies in the search of finding more and better predictive markers of immunotherapy benefit in N2 disease [64]. 

#### 5.3.3. Circulating Tumor DNA (ctDNA)

The detection of ctDNA in the blood in patients with NSCLC may be used for the detection of actionable genomic alterations (i.e., mostly at diagnosis), but also as a means in which to detect potential micrometastatic disease in the early-stage setting. This surrogate of “minimal residual disease” (MRD) could be very useful for patients with N2 disease. The prognostic value of MRD is well-known in leukemias and is now being more and more recognized in patients with solid tumors and may even be used for (de-)escalation strategies [65]. In lung cancer, it has been shown that among patients with EGFR-mutant tumors, baseline ctDNA-positive or MRD-positive status (before and/or after surgery) were associated with poor DFS in curative resected stages I to IIIA [66]. Therefore, its use had been explored in recent prospective trials.

For patients with EGFR-mutant tumors, a recent analysis of ctDNA levels in the ADAURA trial was performed. Around 34% of the patients in both arms had tissue samples to produce MRD panels (comprised of ≤50 tumor-specific variants, based on whole exome sequencing of the resected tumor tissue). Around 4% of cases in the osimertinib and 12% in the placebo groups were MRD-positive at baseline. Then, four out of five patients in the osimertinib group became MRD-negative during treatment, where none of the 13 patients in the placebo group achieved this. During treatment, MRD detection had a clinical sensitivity of 65% and specificity of 95% to detect recurrence, preceding a DFS event by a median of 4.7 months (95%CI, 2.2–5.6) across both arms of treatment. Interestingly, in the osimertinib arm, 58% of the MRD/DFS events detected occurred within 12 months of stopping osimertinib. These findings support the idea that MRD detection could possibly help determine which patients would benefit from extended adjuvant osimertinib treatment [67].

Additionally, three peri-operative chemo-immunotherapy trials have evaluated the use of ctDNA as a monitoring tool during neoadjuvant treatment. In the Checkmate-816, ctDNA clearance could be evaluated in 89 patients before surgery. ctDNA clearance was higher in the chemotherapy-immunotherapy group (56%, 95% CI 40–71) than in the chemotherapy alone group (35%, 95% CI 21–51). Patients in the combination group who achieved ctDNA clearance had a numerically higher EFS versus those who did not achieve ctDNA clearance (not reached versus 18.9 months, respectively), although this was not significant (HR 0.60, 95%CI, 0.20–1.82). In the combination group, there was also a higher rate of pCR in those who achieved ctDNA clearance (46%, 95% CI, 26–67) versus those who did not (0%, 95% CI, 0–18) [35]. 

In the NADIM II trial, ctDNA was measured before and after neoadjuvant treatment. Pretreatment levels were correlated with tumor size, but we have no data regarding its correlation with N status. Patients with pre-treatment ctDNA levels < 5% had a longer DFS and OS in comparison to those with ctDNA ≥ 5% (HR 0.38, 95%CI 0.15–0.95 and HR 0.22, 95% CI, 0.07–0.64, respectively). The ctDNA-negative rate after neoadjuvant treatment was also higher in the experimental group (67% versus 44%) [47]. 

In the AEGEAN trial, ctDNA plasma samples were collected prior to each cycle of neoadjuvant treatment and before surgery. ctDNA detection at the baseline was generally higher in patients with squamous vs. non-squamous histology (98% vs. 85%), higher stage disease (IIIA, IIIB [97%, 96%]) vs. II [82%]), or N2 disease (N2 [96%] vs. N0, N1 [83%, 93%]) [68]. In both treatment arms, decreases in ctDNA were observed in both arms as early as the second cycle. By the third cycle, ctDNA levels were lower in patients with pCR/MPR than in those with non-pCR/MPR. After each cycle of treatment, higher ctDNA clearance rates were observed in the durvalumab than in the placebo arm (66% [95% CI 54–77] versus 41% [95% CI, 30–52] before surgery). Interestingly, patients without ctDNA clearance were very unlikely to achieve pCR, with a negative predictive value of >97% after only two cycles in both arms. 

These studies highlight the value of ctDNA as a potential early-response biomarker that correlates with treatment response and a higher likelihood of pCR and MPR when clearance is achieved. Nonetheless, one of the challenges of this approach for patients with non-oncogene addicted tumors is the lower sensitivity of ctDNA tests, especially in patients with non/low-shedding tumors, which could lead to false negative results, even in the presence of micrometastatic disease. On the other hand, the absence of ctDNA clearance could potentially be used to identify patients with N2 tumors that are not responding to chemo-immunotherapy and may therefore benefit from an escalation strategy (e.g., double immunotherapy) and/or be preferentially treated with other multi-modality treatments such as chemo-radiotherapy. 

## 6. Conclusions

The management of stage III NSCLC remains a complex and evolving field due to the wide range of tumor sizes, locations, and lymph node involvement. Recent trials have highlighted the survival benefit of incorporating immunotherapy and targeted agents in the treatment of N2-positive tumors, sparking important debates about defining subsets of N2 disease, diagnostic modalities, and optimal treatment options. The heterogeneity of N2 disease and the lack of a universal definition of resectability contribute to the uncertainty surrounding the optimal treatment approach for these patients. However, it is widely accepted that systemic treatment such as chemotherapy, immunotherapy, or targeted therapy should be considered for patients with N2 involvement due to their high risk of relapse.

Despite the challenges posed by the heterogeneity of N2 disease and the lack of consensus on the role of surgery, ongoing research and clinical trials are expanding our understanding of the optimal treatment strategies for these patients, paving the way for improved outcomes in the future. It is vital to discuss every case in a multidisciplinary tumor board and to consider the patients’ preferences, in order to provide the best tailored diagnostic and multimodal treatment for each patient according to their disease characteristics, comorbidities, desires, and expected benefits. 

## Figures and Tables

**Table 3:** Clinical trials in the perioperative setting—outcomes.

		pCR Rate—% (95% CI)		EFS/DFS/PFS—Median (95% CI) & HR (95% CI)			OS—Median (95% CI) & HR (95% CI)		Surgical Outcomes	
Trial Name	Treatment Arms	All Patients	N2/Stage III *	End-Point	All Patients	N2/Stage III *	All Patients	N2/Stage III *	R0 §	Did Not Complete Surgery
**AEGEAN [5]**	CT + Durvalumab	17.2% (13.5–21.5)	**All N2**: NA **Single-N2**: 18.4% (12.4–25.8) **Multi-N2**: 8.8 (1.9–23.7)	EFS	NR (31.9-NR) vs. 25.9 (18.9-NR) HR 0.68 (0.53–0.88)	**All N2**: NA **Single-N2:** NR (NR-NR) vs. 22.8 (12.6-NR) HR 0.61 (0.39-0.94) **Multi-N2**: 31.9 (9.3-NR) vs. 12.2 (7.2-NR) HR 0.69 (0.33–1.38)	NA	NA	950%	22%
	CT + Placebo	4.3% (2.5–6.9)	**All N2**: NA **Single-N2:** 4.5% (1.7–9.6) **Multi-N2:** 5.0% (0.6-16.9)				NA	NA	91%	23%
**KEYNOTE 671 [6,48]**	CT + Pembrolizumab	18.1% (14.5–22.3)	NA	EFS	47.2 (32.9-NR) vs. 18.3 (14.8–22.1) HR 0.59 (0.48–0.72)	**All N2:** NA. HR 0.63 (0.48–0.82)	NR (NR-NR) vs. 52.4 (45.7–NR) HR 0.72 (0.56–0.93)	**All N2**: NA. HR 0.74 (0.52–1.07)	92%	18%
	CT + Placebo	4.0% (2.3–6.4)							840%	21%
**Checkmate 77T [7]**	CT + Nivolumab	25.3% (19.8–31.5)	**All N2**: 22.0% (14.0–31.9) **Single-N2**: 18.6% (9.7–30.9) **Multi-N2**: 29.0% (14.2–48.0)	EFS	NR (28.9-NR) vs. 18.4 (13.6–28.1) HR 0.58 (0.42–0.81)	**All N2**: 30.2 (26.9-NR) vs. 10.0 (8.1–15.1) HR 0.46 (0.30–0.70) **Single-N2:** 30.2 (26.9-NR) vs. 10.0 (8.1–15.1) HR 0.49 (0.29–0.84) **Multi-N2:** NR (13.2-NR) vs. 10.0 (8.0–18.8) HR 0.43 (0.21–0.88)	NA	NA	89%	22%
	CT + Placebo	4.7% (2.4–8.3)	**All N2**: 5.6% (1.8–12.5) **Single N2**: 7.5% (2.1–18.2) **Multi-N2**: 2.7% (0.1–14.2)				NA	NA	90%	23%
**Neotorch [8]**	CT + Toripalimab	24.8% (19.0–31.3)	**Stage III:** 24.8%	EFS	NE (24.4-NE) vs. 15.1 (10.6–21.9) HR 0.40 (0.28–0.57)	**Stage III:** NE (NE-NE) vs. 15.5 (9.9–NE) HR 0.40 (0.27–0.57)	NE (NE-NE) vs. 30.4 (29.2-NE) HR 0.62 (0.38–0.999)	NA	96%	NA
	CT + Placebo	1.0% (0.1%–3.5%	**Stage III:** 1.0%						93%	NA
**Rationale 315 [9]**	CT + Tislelizumab	40.7%	NA	EFS	NR vs. NR HR 0.56 (0.40–0.79)	**Stage III**: NR (29.6-NE) vs. 19.8 (13.1-NE) HR 0.62 (0.42–0.94)	NR (NR-NR) vs. NR (35.0-NE) HR 0.62 (0.39–0.98)	NA	NA	16%
	CT + Placebo	5.7%	NA					NA	NA	24%
**NADIM II [47]**	CT + Nivolumab	37%	**Single-N2†:** 42.1% (19.9–64.3) **Multi-N2**: 36.4% (16.3–56.5)	PFS	NR (27.6–NR) vs. 15.4 (10.6–NR) HR 0.47 95% CI 0.25–0.88	**All N2: NA** **Single N2:** NA. HR 2.43 (0.3–19.46) **Multi-N2**: NA. HR 0.39 (0.16–0.94)	NR (33.5-NR) vs. NR (21.1-NR) HR 0.43 (0.19–0.98)	**All N2: NA** **Single-N2:** NA. HR NE **Multi-N2:** NA. HR 0.30 (0.09–1.07)	94%	7%
	CT + BSC	7%	**Single-N2†:** 0% (0–0) **Multi-N2**: 10% (-8.6–28.6)						85%	31%

BSC: best supportive care; CI: confidence interval; CT: chemotherapy; DFS: disease-free survival; EFS: event-free survival; HR: hazard ratio; NA: not available; NE: Not estimable; NR: not reached; ORR: objective response rate; OS: overall survival; pCR: pathological complete response; PFS: progression-free survival. * We preferentially presented the data on N2 outcomes—if these were not available, then we presented the data on stage III outcomes (as it comprises patients with tumors with N2 involvement). † Based on a total of 17 patients who achieved pCR. § Based on the patients that went into surgery.
